# ASO Author Reflections: Understanding the Costs of Major Surgical Intervention

**DOI:** 10.1245/s10434-024-16377-4

**Published:** 2024-10-22

**Authors:** Charles W. G. Risbey, Kilian G. M. Brown, Michael J. Solomon, Kate E. McBride, Daniel Steffens

**Affiliations:** 1 Surgical Outcomes Research Centre (SOuRCe), Royal Prince Alfred Hospital, Camperdown, Sydney, Australia; 2 Faculty of Medicine and Health, Central Clinical School, The University of Sydney, Sydney, Australia; 3 Department of Colorectal Surgery, Royal Prince Alfred Hospital, Sydney, Australia; 4 RPA Institute of Academic Surgery, Royal Prince Alfred Hospital, Sydney, Australia

## Past

The cost of healthcare is rising in Australia and the USA.^1,2^ In 2021–2022, the total health expenditure in Australia was $241.3 billion AUD, an increase of 6% from 2020–2021.^1^ Both nominal and real total health expenditure has increased annually over the decade leading to 2021–2022 in Australia, with healthcare adjusted inflation exceeding general inflation over the same period.^1^ Indeed, in 2021–2022, the ratio of health spending to total government expenses in Australia was 17.2%, higher than at any time throughout the preceding decade.^1^ Similar trends are present in the USA, where total health expenditure has been rising annually over the same period to $4.5 trillion USD in 2022.^2^ Consequently, robust healthcare expenditure data are needed to guide future healthcare spending.^3^

## Present

Complex surgical interventions, including pelvic exenteration (PE), are considered the main treatment option for selected patients with advanced pelvic malignancy.^4^ While PE can provide a curative treatment option for these patients, the admission costs associated with the procedure are significant, typically in excess of $100,000 AUD, reflecting the requirement for specialist multidisciplinary input and risk of significant postoperative morbidity.^3^ However, despite oncological, survival, and quality of life benefits associated with advanced surgical procedures such as PE, funding to support highly specialized surgical referral units is often inadequate, with units dependent on supplemental support to function.^4,5^

## Future

### Strategies for Sustainable Funding and Efficiency

It is evident that significant funding is required to support highly specialized surgical units routinely performing PE and other complex oncological procedures.^3^ To maintain high-volume, specialized surgical referral units, it is essential that adequate funding is allocated to alleviate the reliance on supplementary funding. This stable funding base would ensure consistent delivery of these critical services. As our understanding of cost drivers in specialized surgical units improve, opportunities arise to enhance efficiency without compromising the quality of care. Additionally, implementing strategies such as prehabilitation programs can potentially decrease costs, further optimizing patient outcomes and resource utilization.^6^
